# Design and fabrication of crack-junctions

**DOI:** 10.1038/micronano.2017.42

**Published:** 2017-10-23

**Authors:** Valentin Dubois, Frank Niklaus, Göran Stemme

**Affiliations:** 1 KTH Royal Institute of Technology, Micro and Nanosystem, Osquldas väg 10, Stockholm 100 44, Sweden

**Keywords:** arrays, crack-junctions, lithography, nanofabrication, nanogap electrodes, notches, optimization, tunneling junctions

## Abstract

Nanogap electrodes consist of pairs of electrically conducting tips that exhibit nanoscale gaps. They are building blocks for a variety of applications in quantum electronics, nanophotonics, plasmonics, nanopore sequencing, molecular electronics, and molecular sensing. Crack-junctions (CJs) constitute a new class of nanogap electrodes that are formed by controlled fracture of suspended bridge structures fabricated in an electrically conducting thin film under residual tensile stress. Key advantages of the CJ methodology over alternative technologies are that CJs can be fabricated with wafer-scale processes, and that the width of each individual nanogap can be precisely controlled in a range from <2 to >100 nm. While the realization of CJs has been demonstrated in initial experiments, the impact of the different design parameters on the resulting CJs has not yet been studied. Here we investigate the influence of design parameters such as the dimensions and shape of the notches, the length of the electrode-bridge and the design of the anchors, on the formation and propagation of cracks and on the resulting features of the CJs. We verify that the design criteria yields accurate prediction of crack formation in electrode-bridges featuring a beam width of 280 nm and beam lengths ranging from 1 to 1.8 μm. We further present design as well as experimental guidelines for the fabrication of CJs and propose an approach to initiate crack formation after release etching of the suspended electrode-bridge, thereby enabling the realization of CJs with pristine electrode surfaces.

## Introduction

Electrode-nanogap-electrode structures, so-called nanogap electrodes^1^ or electronic nanogaps^2^, are used in nanoelectronics, nanophotonics, plasmonics, nanopore sequencing, molecular electronics, and molecular sensing. They provide a powerful test-bed for mesoscopic physics^3–7^. Electronic nanogaps with gap-widths in the sub-5 nm range are particularly interesting as they are suitable for embedding and probing molecules to investigate electron transport mechanisms and strong light-matter interactions on a molecular-level. In addition, electrodes separated by sub-2 nm wide gaps can be operated as tunneling junctions that have manifold potential applications such as DNA sequencing, quantum computing, RF and optical emitters^4,8–11^. However, it remains extremely challenging to fabricate large numbers of nanogap electrodes featuring gap-widths below 5 nm in a reliable and efficient way. Accurately producing sub-5 nm wide gaps requires a patterning resolution of a few atomic layers, which is a great technological challenge even if only a few devices are to be realized^12^. Existing nanogap fabrication techniques include mask-defined etching processes^13^, layer-defined sacrificial etching processes^14^, material-growth processes^1,15^, self-assembly processes^7,16^, and the break junction (BJ) technique^17–19^. Each of these approaches suffers from severe drawbacks, including limited process control, limited dimensional accuracy, limited process scalability and risk of residual contaminants inside the nanogaps. A novel concept was proposed and recently demonstrated for fabricating crack-defined nanogap electrodes^2,20,21^, so-called crak-junctions (CJs). CJs can be fabricated on wafer-level in very large numbers and feature gap-widths that can be precisely defined in a range from below 2 to 100 nm and above. CJs are formed by controlled fracture of pre-strained electrode-bridges fabricated in a thin electrically conducting film, thereby forming electrode pairs that exhibit nanoscale gaps. A key advantage of the CJ methodology is that while the electrode-bridges are defined lithographically, the resulting crack-defined gaps are self-generated and have predictable atomic-scale dimensions that cannot be realized with conventional state-of-the-art nanofabrication technologies. CJs also display unique properties such as the possibility to realize high aspect ratios between gap-height and gap-width, and perfectly matching electrode surfaces^2^. Other works on the characterization of the mechanical properties of ductile and brittle thin films have also been reported using release of internal stress in suspended structures to generate various controlled stress loading situations^22–24^. CJs use a similar approach but for inducing cracks in brittle, electrically conducting thin films and defining nanogaps with controlled widths. Successful fabrication of CJs requires precise control of the formation and propagation processes of the crack by utilizing well-designed stress-concentrating structures. While the realization of CJs has been demonstrated in initial experiments^2,21^, the impact of the different design parameters of CJs on the resulting cracks has not yet been thoroughly investigated. Specifically, we analyze theoretically and verify experimentally the influence of the dimensions and shape of the stress concentration structures (notches) in the electrode-bridge, the beam length of the electrode-bridge, and the anchor design of the electrode-bridge, on the formation and propagation of the crack. In addition, we present an approach to initiate crack formation by substrate cooling after the electrode-bridges are release etched, as opposed to forming the cracks during the release etching process, thereby enabling the realization of CJs featuring pristine electrode surfaces.

## Materials and methods

### CJ fabrication methodology

**Figure 1:**
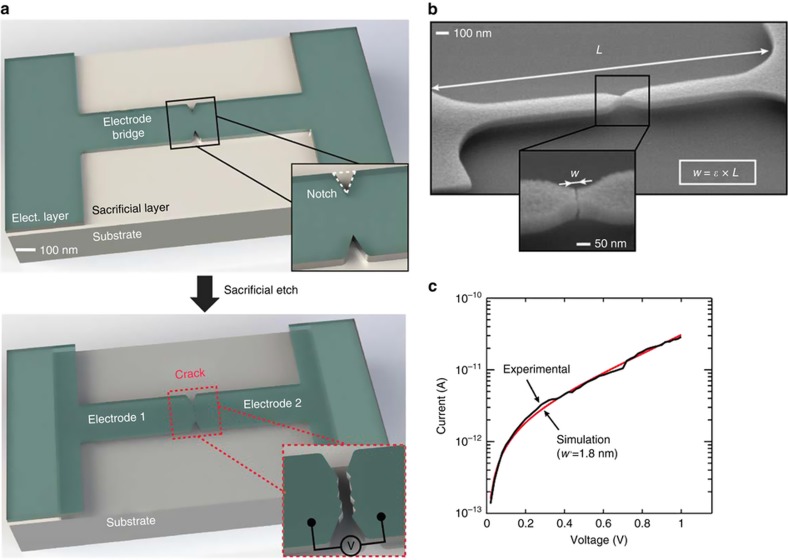
(**a**) Schematics illustrating the crack-junction (CJ) methodology. Top panel: patterned electrode layer before release etching. The electrode layer is brittle and under internal tensile stress at room temperature. Bottom panel: CJ after release etching and cracking of the electrode-bridge, thus defining a gap separating the electrodes. (**b**) Scanning electron microscope (SEM) image shows a 2.7 μm long cracked electrode-bridge featuring suspended titanium nitride (TiN) electrodes and a 10-nm wide gap (inset), illustrating that, for CJs, *w* is proportional to *L* by the factor *ε*, which is the stored elastic strain of the electrode layer. (**c**) I–V plot of a TiN CJ displaying electron tunneling behavior. The Simmons formula^27^ was used to fit the experimental curve, revealing an effective gap-width *w*’ of 1.8 nm for this CJ. For the fitting, an electrode work-function of 4.4 eV is used.

The CJ fabrication methodology is conceptually illustrated in Figure 1a. The process starts by depositing an electrically conductive thin film, called here electrode layer, on top of a sacrificial layer on a substrate. The electrode layer must exhibit residual internal tensile stress and brittle fracture behavior. A notched electrode-bridge structure is patterned in the electrode layer (Figure 1a, top panel) using a resist mask and plasma etching. The sacrificial layer is then selectively etched away using isotropic chemical etching (Figure 1a, bottom panel). During this step, the electrode-bridge is detached from the substrate and the stored elastic strain redistributes to maintain equilibrium of stresses. This causes the build-up of stress at the notch of the electrode-bridge. Once the local stress level at the notch overcomes the strength of the electrode material, a crack is initiated at the notch. This results in a fracture across the notched constriction (neck) of the electrode-bridge, contraction of the free-standing electrodes in opposite directions, and the formation of a nanoscale gap that is separating the electrodes, as illustrated in Figure 1a (inset of bottom panel). The internal stress initially stored in the electrode-bridge is converted to an accurate and predictable self-generated retraction of the electrodes after crack formation. The length *L* of the suspended part of the electrode-bridge defines the width *w* of the resulting nanogap, whereby short electrode-bridges yield small gap-widths. As shown in Figure 1b, *L* and *w* are proportional to each other with the stored elastic strain of the electrode layer *ε* as proportionality constant with^2^:

(1)w=ε×L,

where *ε* is equal to the internal stress of the electrode layer *s* divided by its Young’s modulus *E*:

(2)ε=s/E.

Thus, the CJ methodology is based on the down-conversion of the micrometer-scale length of the electrode-bridge defined by standard lithographic patterning, into a precisely controlled nanometer-scale displacement of the tip of the electrodes to define the gap, whereby the attenuation factor is the elastic strain *ε* of the electrode layer. When the length of the electrode-bridges is below 1 μm, the effective inter-electrode separations can be below 3 nm, thereby resulting in CJs that exhibit electron tunneling characteristics, as shown in Figure 1c. This entire process to form a single CJ can easily be applied to large numbers of CJs on a substrate simply by pre-patterning lithographically many electrode-bridges in the same electrode layer on chip or wafer-level. As the internal tensile stress is uniformly distributed in the electrode layer over the entire substrate, any pre-patterned electrode-bridge will automatically form a crack. This process is also compatible with integration on top of complementary metal oxide semiconductor (CMOS) circuitry wafers^25^.

### Substrate preparation and baseline CJ fabrication

In all experiments, the starting substrate consists of a 525 μm-thick and 100 mm diameter p-doped (100) silicon wafer, covered by a two-layer stack composed of a 70 nm layer of aluminum oxide (Al_2_O_3_), as the sacrificial layer, and a 70 nm layer of titanium nitride (TiN), as the electrode layer, both deposited in an atomic layer deposition (ALD) tool (Beneq TFS 200). The Al_2_O_3_ is deposited at a temperature of 200 °C in 700 cycles using trimethylaluminum (TMA, pulse time 70 ms, purge time 500 ms) and water (H_2_O, pulse time 175 ms, purge time 750 ms) as precursors. The TiN is deposited at a temperature of 350 °C in 2000 cycles using titanium tetrachloride (TiCl_4_, pulse time 150 ms, purge time 500 ms) and ammonia (NH_3_, pulse time 1 s, purge time 1 s) as precursors. After depositing the stack of thin films, the wafer is cleaved into dies with a size of 1 cm×1 cm. The dies are then spin-coated with a positive e-beam photoresist (ZEP7000, Zeon Chemicals, Japan) and baked at 170 °C for 3 min on a hot plate. The resist thickness is 180 nm after baking. The notched electrode-bridges are defined in the photoresist by exposure in a Raith e-beam system at 25 keV acceleration voltage with an area step size of 8 nm and an area dose of 84 μAs cm^−2^. The exposed resist is developed in a p-Xylene solution for 100 s and in a Methyl isobutyl ketone (MIBK) solution for 10 s and then dried with a nitrogen gun. The resist-defined pattern is then transferred into the TiN by dry anisotropic plasma etching (Applied Materials Precision 5000 Etcher) at a chamber pressure of 200 mTorr and RF power of 600 W in a mixture of boron trichloride (BCl_3_) at 40 s.c.c.m. flow, chlorine (Cl_2_) at 15 s.c.c.m. flow, nitrogen (N_2_) at 15 s.c.c.m. flow, and tetrafluoromethane/oxygen (CF_4_/O_2_) at 15 s.c.c.m. flow. The resist mask is subsequently removed with remover (Microresist, rem-700) at 60 °C in an ultrasonic bath for 10 min. The smallest features implemented in the resist layer are the notches, which are resolved with dimensions down to 50 nm. The notch patterns in this study were generated using e-beam lithography, but it is in principle also possible to realize the notch patterns by using state-of-the-art stepper photolithography systems for high-throughput wafer-scale fabrication. At this point, the electrode-bridges have been patterned but are still resting on the sacrificial material, as shown in Figure 1a, top panel. To form the CJs, as shown in Figure 1a, bottom panel, the electrode-bridges are released by sacrificial isotropic etching of the Al_2_O_3_ layer in a KOH bath at room temperature for 20 min. Cracks in the electrode-bridges are typically formed during this sacrificial etching step. Thereafter the devices are dried using critical point drying (BalTec CPD 408), thus avoiding stiction of the suspended electrodes to the substrate. TiN was chosen as an electrode material because of its attractive structural, plasmonic, and superconducting properties, which makes it a very promising electrode material for a variety of nanogap-based devices and applications. However, since the crack follows the grain boundaries of polycrystalline TiN, the cracked TiN surfaces have a non-planar geometry which affects the gap-widths of the resulting CJs (Supplementary Information).

### Measurement of the internal stress

The internal stress of a TiN thin film deposited on a 525 μm thick p-doped single-crystalline silicon wafer (100) was measured using a surface profiler (Tencor-P15). The wafer curvature was recorded before and after ALD deposition of the TiN film using identical deposition parameters as used in the baseline CJ fabrication process. The measured wafer curvature revealed a constant internal stress in the TiN film of 1.60±0.05 GPa at time points of 20 min, 1 h, 1 day and 1 week after the material deposition, respectively. Thus, for a time period in the range of days, the elapsed time between the deposition of the TiN film and the CJ fabrication does not appear as a critical parameter that is inducing variability in our experiments. Considering the measured elastic strain^2^ of *ε*=3.1 nm μm^−1^, the biaxial Young’s modulus of the ALD deposited TiN film can be estimated to *E*’=*σ*/*ε*≈530 GPa, which is consistent with values reported in literature for sputtered TiN^26^. We further investigated the contribution of the baking step of the e-beam resist on stress relaxation in the TiN film and found that after 3 min of baking on a hot plate at 170 °C, the internal stress in the TiN film remained within 1.60±0.05 GPa.

### Electrical characterization

To confirm and demonstrate the realization of tunneling CJs, we performed tunneling measurements of a CJ as shown in Figure 1c. Therefore, single CJs that were electrically connected to 150 μm×150 μm large probing pads were fabricated. The probing pads and the electrical wiring between the probing pads and the CJ electrodes were made of the same TiN layer deposited in the same process step as the CJs electrodes. Thus, no additional metallization layers were necessary for realizing the probing pads and the electrical connections between the probing pads and the CJs. This approach avoids the risk of introducing stress gradients to the CJ electrodes by introducing additional metal layers. Electrical probing of the TiN probing pads was carried out with tungsten carbide tips in a semi-automatic shielded wafer prober (Cascade Microtech 12000). The thin TiN probing pads were sufficiently stable to allow reliable electrical measurements of the CJs. A parameter analyzer (Keithley SCS 4200) and two high-resolution Source Measure Units (SMU) combined with low-noise pre-amplifiers were connected to the probe-tips. The Simmons formula^27^ was used to fit the I–V measurement curve in Figure 1c, revealing an effective gap-width *w*’ of 1.8 nm.

### Modeling and simulation

**Figure 2:**
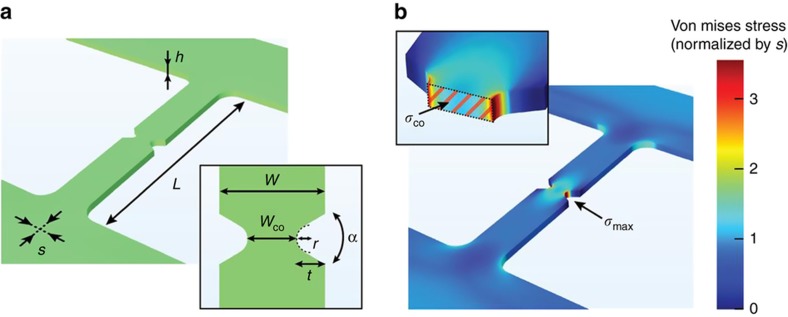
(**a**) Three-dimensional (3D) perspective sketch of an electrode-bridge, detailing the main design parameters of CJs: *h* is the thickness of the electrode layer, *L* is the length of the electrode-bridge, and *s* is the internal tensile stress in the electrode layer. Inset: *W* is the width of the electrode-bridge, *W*_co_ is the width of its notched constriction (neck), *r* is the radius of the notch tip, α is the open angle of the notch, *t* is the notch indent, which is equal to *r* for a semi-circular notch. (**b**) 3D FEM Von Mises stress map of the electrode-bridge shown in (**a**), where *σ*_max_ is the highest stress in a CJ, located at the notched edges. In this design, for which *r*=37.5 nm, *α*=60°, and *t*=75 nm, it is found that *σ*_max_=3.4*s*. Inset: *σ*_co_ is the average stress at the cross-section of the neck.

To compute the maximum stress at the notched constriction, finite element method (FEM) simulations were performed using the software package COMSOL Multiphysics 5.3 (Stockholm, Sweden) using a three-dimensional (3D) geometry model that includes the undercut profile in the sacrificial layer. The model was synchronized with, and imported from SolidWorks Corps using the LiveLink interface. Considering the symmetries in the structure, it is sufficient to simulate only a quarter of the electrode-bridge. The complete mesh of a quarter of the representative CJ in Figure 2 contained 41486 domain elements, 7678 boundary elements, and 599 edge elements (see Supplementary Information for details on the constitutive model).

## Results and discussion

### Theoretical considerations on the distribution of stress in CJs.

A detailed sketch of a CJ with the different design parameters is shown in Figure 2a. For our analysis, we keep the thickness *h* of the electrode layer and the width *W* of the electrode-bridge constant to 70 and 280 nm, respectively. We also assume that the electrode material features brittle mechanical properties, and that the release etching has no other effect than releasing the internal tensile stress in the electrode material. The design of the electrode-bridge used to form the CJ is conceptually comparable to a double-notched, double-clamped beam. Initially under residual tensile stress, the electrode-bridge cannot contract in the beam direction to relieve the internal stress during the release etching step. Thus, the stress persists and can be viewed as a force applied on the beam, pulling it longitudinally. A 3D stress map of an electrode-bridge with *L*=1.7 μm and *W*=280 nm is shown in Figure 2b, where we model the V-shaped notches since it is similar to the notch geometry that results from the e-beam lithography patterning in our experiments. Under the correct conditions, the stress level at the notched edges of the beam is sufficiently high to form a crack.

As shown in Figure 2b, the highest stress in the electrode-bridge *σ*_max_ is localized at the notched edges of the neck, thereby localizing the crack initiation at the notched constriction of the electrode-bridge. There are two intertwined components contributing to the high stresses triggering the formation of the crack: the constriction effect and the notch effect. First, the constriction effect relates to the accumulation of stress caused by the narrowing of the electrode-bridge beam from an initial width of *W* to the width of the notched constriction *W*_co_. For an electrode-bridge with a uniform thickness that is loaded on its outer edges by a stress *s*, the equation governing this component is obtained by the equilibrium condition of the forces along the electrode-bridge:

(3)σco=σbridge×Sbridge/Sco=W/Wco×s,

where *σ*_co_ and *S*_co_ are the average stress and the cross-sectional area at the constriction of the electrode-bridge, respectively, and *σ*_bridge_ and *S*_bridge_ are the stress and the cross-sectional area of the electrode-bridge, respectively.

Second, the notch effect relates to the severity of the localization of the stress induced by the geometrical shape of the notches that outline the edges of the neck, whereby acute, and sharp, notched edges yield higher stress concentrations than obtuse and rounded ones. This effect is superimposed on the constriction effect and can be quantified by the net theoretical stress concentration factor *K*_tn_ (Ref. 28). Equation (3) thus becomes:

(4)σmax=Ktn×W/Wco×s.

where *σ*_max_ is the maximum occurring localized stress at the notches.

To initiate a crack and successfully form CJs, the electrode-bridges and notches should be designed such that *σ*_max_ overcomes the fracture strength of the electrode material σ*_max_. For the specific electrode-bridge design shown in Figure 2, the calculated *σ*_max_ is 3.4 times the internal tensile stress *s* of the electrode layer. We will see that this is sufficient to initiate the fracture in electrode-bridges made of TiN in our experiments, but may or may not be for other electrode materials. A high yield of crack formation, which is desirable for fabricating CJs in a reliable way, thus involves adequate design of the electrode-bridges and notches to achieve *K*_tn_×*W*/*W*_co_×*s*>σ*_max_. To satisfy this relation, we can select and design: (i) the material, and aim at minimizing the fracture strength σ*_max_, (ii) the fabrication process, and aim at maximizing the internal stress *s*, (iii) the design of the electrode-bridges and notches, and aim at maximizing the notch effect *K*_tn_ and the constriction effect *W*/*W*_neck_. In this section, we will focus on (iii).

The guidelines to design notches in a way so that they promote crack formation are simple in theory as it requires making the notches as sharp and acute as possible, thus minimizing *r* and *α*, and maximizing *t*. In practice, however, it is not possible to freely adjust these geometrical parameters since lithography and pattern-transfer steps will severely impede accurate reproduction of the notch geometry. E-beam lithography could in theory resolve U-shaped notches, while optical lithography would produce more V-shaped notches with a larger notch radius *r*. *K*_tn_ can be accurately estimated for both U and V-shaped notches using textbook tables^28^. Knowing *K*_tn_ and the geometry of the electrode-bridge, the maximum occurring localized stress at the notches *σ*_max_ can be derived using Equation (4), and if *σ*_max_>σ*_max_, the CJ should form successfully.

To illustrate the trend typically found for lithographically defined electrode-bridges, we take the example of semi-circular notches, for which *t*=*r* and *W*_co_=*W*−2*r*, and find^28^:

(5)σmax/s≈3×(1–11–1.1(2r/W)+0.33(2r/W)2+0.13(2r/W)3)/(1−2r/W).

**Figure 3:**
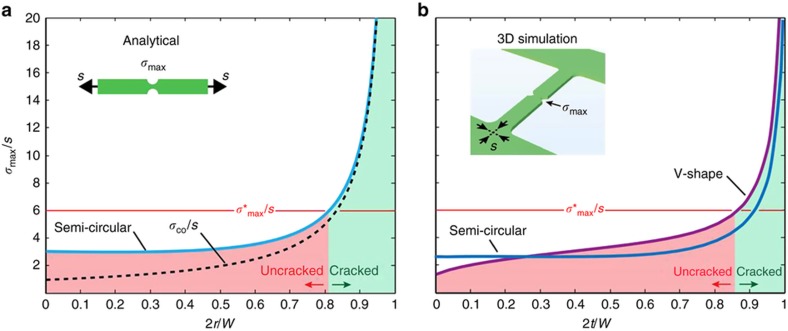
(**a**) Plot of the maximum stress *σ*_max_ normalized by s using the stress concentration factor formula^28^ for a semi-circular notch design (blue curve). The constriction effect of Equation (3) (black dashed curve) was added as reference. (**b**) Plots of the maximum stress *σ*_max_ normalized by s based on 3D FEM modeling for the V-shaped notched electrode-bridge shown in Figure 2 (purple curve, for varying notch indent *t*, while maintaining the beam width *W* and notch radius *r* constant at 280 and 75 nm, respectively), and for semi-circular notches (blue curve, for varying notch indent *t*, with *t*=*r*, while maintaining the beam width *W* constant at 280 nm). To achieve reliable crack formation, the electrode-bridge of the CJ must be designed in a way that the maximum occurring localized stress at the notches is higher than the fracture strength of the electrode material: *σ*_max_>σ*_max_ (green area). The placement at σ*_max_/*s* on this graph at 6 may be lower or higher in practice, depending on the material chosen as electrode.

Equation (5) is plotted in Figure 3a (blue curve) and shows that *σ*_max_ increases from 3 *s* towards infinity when 2*r*/*W* increases from 0 (small notches or comparatively wide necks of the electrode-bridge) to 1 (large notches or comparatively narrow necks of the electrode-bridge), as expected from the gradual narrowing of the neck. Electrode-bridges that exhibit a maximum occurring localized stress at the notches *σ*_max_ smaller than the electrode fracture strength σ*_max_ (red area) remain uncracked, while electrode-bridges that exhibit a notch design with *σ*_max_ larger than σ*_max_ (green area) form a crack. Figure 3a also reveals that, for semi-circular notches, the maximum occurring localized stress at the notches *σ*_max_ is constant over a wide range of values, up until 2*r*/*W* reaches about 0.5. This is because an increase in notch radius (decreasing notch effect) is necessarily accompanied with a narrowing of the constriction (increasing constriction effect). Overall, for semi-circular notches, the constriction and notch effects compensate each other until 2*r*/*W*>0.5, which is when the constriction effect begins to dominate.

This textbook approach helps in obtaining a quick estimation of *σ*_max_ from a given notch geometry. It does not, however, reproduce perfectly the actual stress situation in the electrode-bridge. In our experiments, the electrode layer is under residual tensile stress, which results in electrode-bridges with anchored extremities and exhibiting an in-plane biaxial tensile stress state, instead of the uniaxial loading for the analytical *K*_tn_. Also, the 3D boundary asymmetries existing in a CJ are not accounted for in an analytical *K*_tn_. For a precise quantitative evaluation of *σ*_max_, a 3D FEM approach is more suitable. In Figure 3b, we plotted the results of 3D simulations of the maximum localized stress for the specific electrode-bridge design shown in Figure 2 for semi-circular notches (blue curve) and V-shaped notches (purple curve). This investigation reveals small yet evident differences between analytical *K*_tn_ and 3D FEM simulation results for a semi-circular notch design: in the simulation, the plateau is at *σ*_max_/*s*=2.6, instead of *σ*_max_/*s*=3 for the analytical *K*_tn_, and the contribution of the constriction effect is delayed until 2*t*/*W* reaches about 0.65, instead of 0.5 for analytical *K*_tn_. A V-shaped notch, on the other hand, develops a steady increase in *σ*_max_/*s* with increasing 2*t*/*W* provided the notch radius remains constant.

### Experimental investigations of the notch effect and the electrode-bridge length in TiN CJs

**Figure 4:**
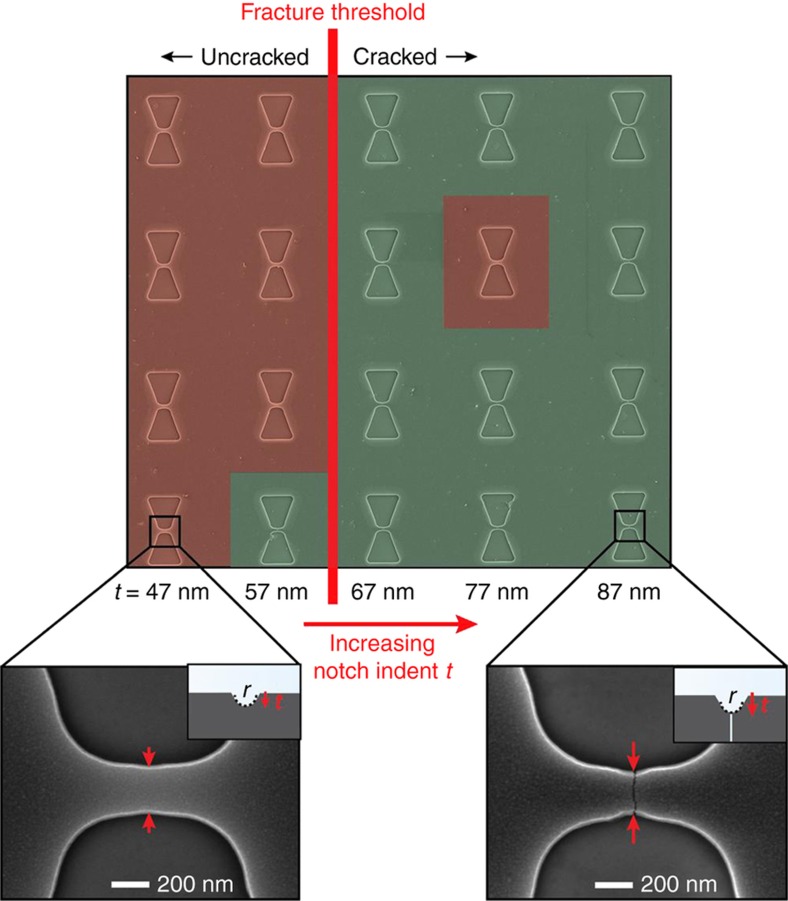
Color-coded SEM image of a CJ matrix with different electrode-bridge designs for evaluating the impact of notch indent *t* on crack formation in the electrode-bridge. As expected from Figure 3b, there exists a critical value of the notch indent *t* that defines a fracture threshold. The red coloring indicates electrode-bridges that are not cracked, while green coloring indicates those that are cracked. Five different CJ designs, corresponding to the five columns of the matrix, were implemented, featuring increasingly large notch indents. To assess reproducibility of the results, four repetitions of each of the five CJ designs are implemented in each column. All CJs feature *W*=280 nm and *L*=1 μm. Because of e-beam lithography patterning in our experiments, the notches have a shallow V-shape, although initially designed as V-notches.

We investigated the influence of the notch effect for a CJ design based on a comparatively short electrode-bridge, that is relevant for fabricating tunneling junctions in TiN. A matrix of CJs was fabricated and each CJ in a row featured an electrode-bridge design with identical *L*=1 μm and *W*=280 nm, but with increasing notch indents *t*, as seen in Figure 4. To assess reproducibility of the results, four repetitions of each of the five CJ designs are implemented in each column. Due to their sub-100 nm size, the notches exhibited a shallow V-shape featuring a gradual increase in notch indent *t*. Inspection of the CJ devices in the matrix after the release etching of the electrode-bridges confirms that there is a fracture threshold that defines a critical value of the notch indent *t* that induces cracking of the bridge structures during release, while electrode-bridges with notch indent below that value typically do not crack. Considering the actual shape of the notches after pattern-transfer, we estimated that *K*_tn_ had to be at least 2 to initiate crack formation in this CJ design for the TiN used here. Thus, other notches featuring sharper features or larger notch indents will automatically provoke fracture. Such feature sizes and geometries are within reach of most electron-beam lithography systems, and many wafer-scale lithography tools such as stepper and nanoimprint, thus indicating that the fabrication of tunneling CJs made in TiN can be realized with wafer-scale processes.

The sharp transition between cracked and uncracked electrode-bridges reveals that it is possible to gain high control over crack formation, allowing us to predict that a specific electrode-bridge and notch design will work, while another will not, as shown in Figure 3. This could be utilized for example, to fabricate an array consisting of CJs that remain uncracked but that are close to fracture. We will later demonstrate that the application of external factors such as controlled substrate cooling and bending, thereby momentarily increasing the tensile stress *σ*_bridge_ in the electrode-bridge, can trigger the fracture event of electrode-bridge designs that were uncracked after the release etching step.

**Figure 5:**
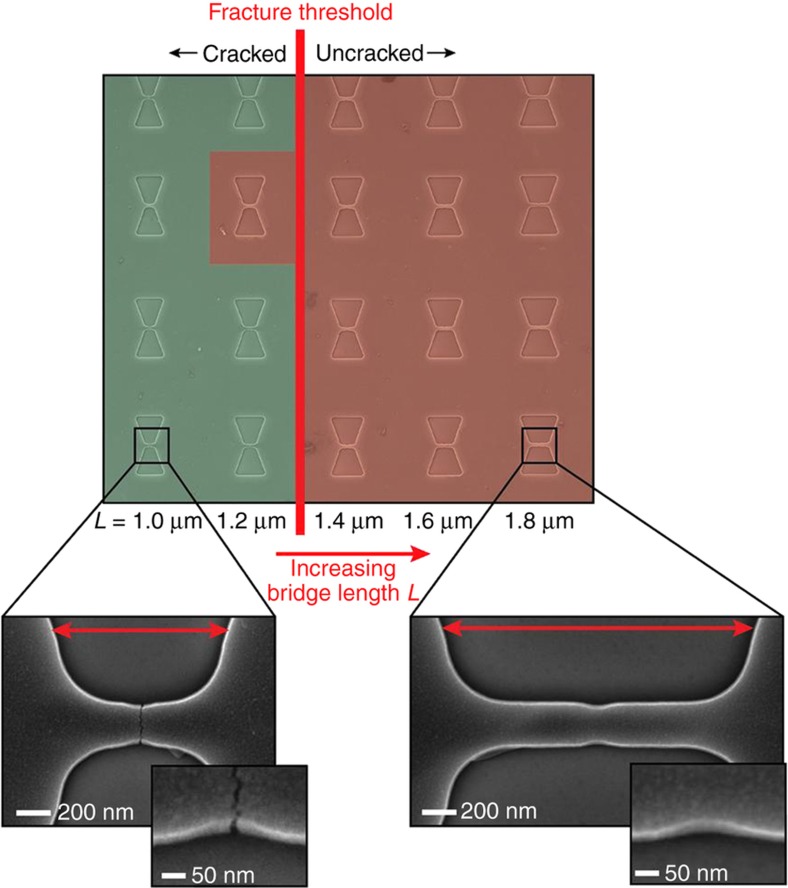
Color-coded SEM image of a CJ matrix for evaluating the impact of the length *L* of the electrode-bridge on crack formation. For a given notch design, there exists a critical value of *L* that defines a fracture threshold. The red coloring indicates electrode-bridges that are not cracked, while green coloring indicates those that are cracked. Five different electrode-bridge designs, corresponding to the five columns of the matrix, were implemented, each featuring an identical notch design but different electrode-bridge length *L*. To assess reproducibility of the results, four repetitions of each of the five CJ designs are implemented in each column. All CJs feature *W*=280 nm and *t*=87 nm. The starting design, at the left-most column of this matrix, is identical to that of the right-most column of the matrix of Figure 4. For this specific design, all eight electrode-bridges present in both matrices have cracked reliably. Although all bridges featured an identical notch design, the SEM insets reveal that the longer bridge exhibit a slightly higher notch radius (i.e., lower *K*_tn_) as compared to the short bridge, which can explain the existence of the fracture threshold caused by changes in bridge length.

However, a notch design that provokes fracture of a specific electrode-bridge design is not necessarily adequate for other electrode-bridge designs. In Figure 5, we have further evaluated the influence of the length *L* for an electrode-bridge from the previous matrix for which the notch effect was sufficiently high to initiate a crack. In this investigation, the geometry of the notch was kept identical while the electrode-bridge length *L* was gradually increased, from 1 to 1.8 μm. We observe that there exists a fracture threshold for which the initially cracked design no longer cracks. This disagrees with the theory of notched beams under uniaxial tension since the cross-sectional tension force along the beam should be independent of the beam length. We performed 3D FEM simulations comparing short and long electrode-bridges under biaxial stress and found no significant difference that could explain such clear dependence on the bridge length. We believe the main explanation for the result is an experimental artifact since we expect a significant difference in exposed area between long and short electrode-bridges, whereby resist for long electrode-bridges receives a significantly larger exposure. The resist over-exposure tends to smear the sharper features of the notches, thus provoking a blunting effect by increasing *r* and *α*, and resulting in a lower *K*_tn_. This hypothesis is supported by scanning electron microscope (SEM) images of the notches after pattern-transfer (see SEM insets in Figure 5). These results highlight the importance of process parameters such as resist exposure and pattern-transfer to obtain reliable and predictable crack formation.

### Influence of anchor design on gap definition and crack propagation in CJs

**Figure 6:**
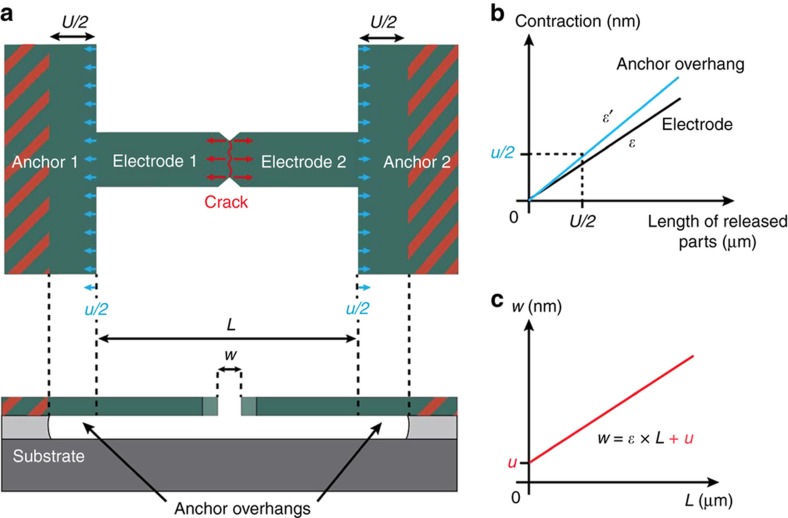
Effect of the anchor overhangs on the definition of the gap-width in a crack-junctions (CJ). (**a**) Schematic top and cross-section views of a CJ showing the different released parts contributing to the definition of the gap-width after crack formation. The presence of anchor overhangs on each side of the electrode-bridge contributes to the total contraction of the cracked electrode surfaces of the CJ. (**b**) Plots of the contraction of each released part of the CJ as a function of their respective length. The elastic strain *ε* of an electrode is slightly lower than that of an anchor overhang *ε’*. (**c**) Plot of the gap-width *w* of a CJ as a function of the length *L* of the electrodes. The presence of the anchor overhangs introduces an intercept *u*.

Anchors have two main effects on the features of CJs. First, anchors affect the definition of the width of the gap since a non-negligible part of the anchors is undercut during the release etching step, as indicated in Figure 6a. Initially under biaxial tensile stress, the anchor overhangs are free to relax in the direction of the beam axis after crack formation, thereby contributing to the total electrode contraction that defines the gap-width, as shown by the blue arrows in Figure 6a. Yet, as the anchor overhangs remain constrained in the direction perpendicular to the beam axis, the overhangs contract per unit length to a different extent (typically larger, due to the Poisson effect) than the electrode parts after crack formation, as shown in Figure 6b. For a given undercut length *U*/2, the linear relation between *L* and *w* for CJs is increased by the total contraction *u* of both anchor overhangs, as indicated in Figure 6c, which may be significant in case of large undercuts. This additional contraction caused by the anchor overhangs should be considered in the design of tunneling junctions as it can easily amount to a few nanometers, which could cause the distance between the cracked electrode surfaces to exceed the direct tunneling range. Nonetheless, the total contraction *u* caused by the anchor overhangs can be predicted with high accuracy by 3D FEM simulations of CJs that account for the geometry and position of the anchor overhangs defined by the undercut.

**Figure 7:**
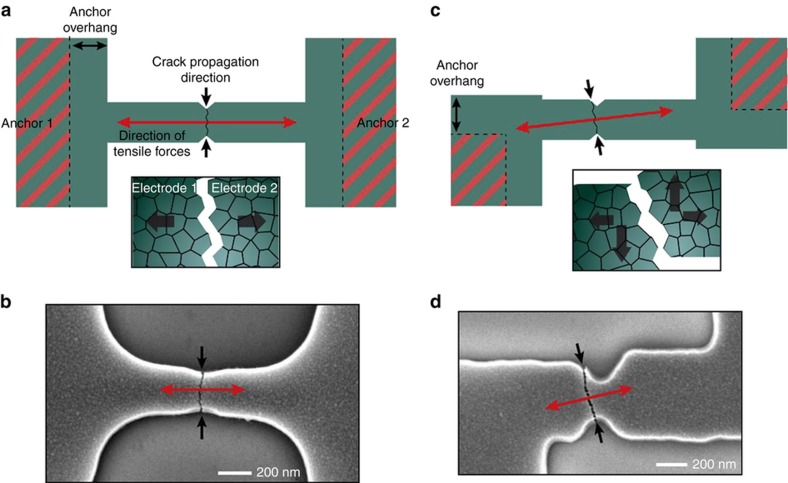
Schematic top views (**a**, **c**) and SEM images (**b**, **d**) illustrating the impact of the placement of the anchors on the direction of the resulting fracture line in the electrode-bridge. In **a** and **c**, the areas marked in red correspond to the anchors of the electrodes, and the insets are schematics of close-ups of the nanoscale topographies of the fractured electrode surfaces, illustrating the effect of electrodes with aligned (**a**) and misaligned (**b**) cracked surfaces. The dimensions of the electrode-bridges in these experiments are *L*=1.2 μm, *W*=0.2 μm, for (**b**) and *L*=1 μm, *W*=0.55 μm, for (**c**), respectively.

Secondly, the anchor design affects the distribution of the stress fields in the electrode-bridge and at the notched constriction of the electrode-bridge, potentially altering the crack path. In the baseline electrode-bridge design shown in all Figures so far, the anchors connecting the electrodes to the substrate are placed symmetrically on the central axis of the electrode-bridge. In this configuration, before fracture, the tensile force acting at the notched constriction of the electrode-bridge is parallel to the central axis of the electrode-bridge, and the crack thus propagates perpendicularly to this axis, as illustrated in Figures 7a and b. In contrast, an electrode-bridge with anchors that are not placed symmetrically with respect to the central axis of the electrode-bridge fractures along a path that is tilted, as illustrated in Figure 7c. An example of a fabricated CJ featuring a tilted crack is shown in Figure 7d. A tilted crack may be detrimental to the functionality of the CJ since the electrodes may contract in directions that are not perpendicular to the crack direction, as shown in Figure 7d, thus leading to misalignment of the otherwise matching electrode topographies. For cracked electrodes exhibiting a surface roughness comparable to the gap-width, misalignment in electrode topographies may even cause undesirable mechanical and electrical contact.

### Additional considerations for facilitating crack formation in CJs

**Figure 8:**
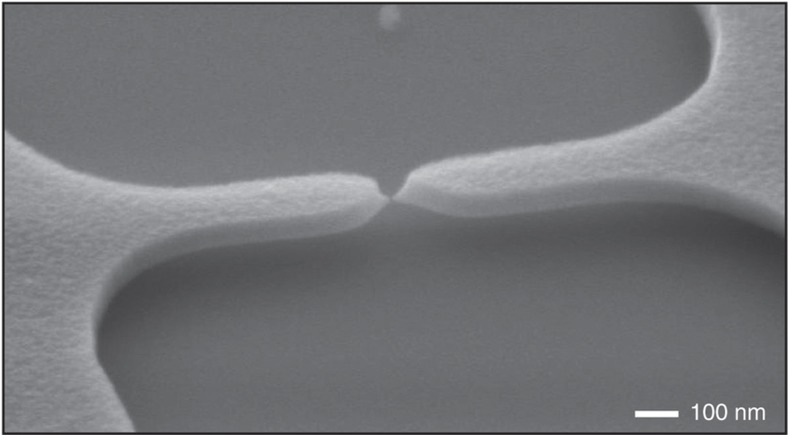
SEM image with a perspective view of a CJ featuring a locally thinned notched constriction. The cracked electrode surfaces facing each other have a cross-sectional area of approximately 10×10 nm^2^, and thus, the nanogap between the electrodes has a volume of approximately 10×10×10 nm^3^. The notched constriction of this CJ was thinned prior to the formation of the CJ by deliberately overexposing the resist polymer in the notch areas during e-beam exposure, thereby obtaining a locally thinner mask after resist development. Thus, during etching of the TiN layer for patterning the electrode-bridge, the locally thinned resist mask is removed during the etching process, which causes partial etching of the top surface of the TiN layer at the notched part of the electrode-bridge. Note the absence of bending of the released TiN electrodes indicating an extremely low out-of-plane stress gradient in the TiN layer.

Because the resolution of the used patterning technology sets a lower limit for the radius of the notch *r*, the notch effect cannot be increased arbitrarily and experiences practical constrains. Conversely, the constriction effect is not limited to the same extent by practical constrains. Thus, initiating cracks can always be facilitated by minimizing the cross-sectional area of the notched constriction *S*_co_ versus the cross-sectional area of the electrode-bridge *S*_bridge_ (Equation (3)). In practice, this can be achieved simply by reducing the width of the notched constriction *W*_co_. Additionally, *S*_co_ can be effectively decreased by locally thinning the electrode layer, as demonstrated in the CJ shown in Figure 8, for which *S*_bridge_/*S*_co_=140.

From a fabrication perspective, the internal tensile stress in the electrode layer *s* is the most important parameter for CJs. First, because *σ*_max_ is proportional to *s* (Equation (4)), increasing *s* will automatically promote crack formation. Second, *s* also impacts the resulting gap-width since *s* is proportional to the elastic strain *ε* (Equation (2)), which connects the gap-width *w* to the length *L* of the electrode-bridge (Equation (1)). This comes with the benefit of reducing the overall footprint of CJs since, for a given gap-width *w*, increasing the internal tensile stress *s* implies reducing the length *L* of the electrode-bridge. While it may be advantageous to maximize *s* from these viewpoints, it should remain within reasonable levels to avoid uncontrolled cracks in the thin electrode layer or extreme warping of the substrate. For the TiN layer used in this work, we, for example, observed catastrophic failure of TiN when a 100 nm-thick TiN layer was deposited on top of a quartz substrate at 350 °C. In this case, the large difference in the coefficients of thermal expansion (CTE) between TiN and quartz caused very high residual tensile stresses after deposition of the TiN. Yet, it may be advantageous to choose a substrate material featuring a low CTE as it typically increases the thermal stress after deposition of the electrode layer. It is also possible to further increase the tensile stress momentarily in the electrode layer by manually warping the substrate, or by cooling the substrate down to below room temperature.

**Figure 9:**
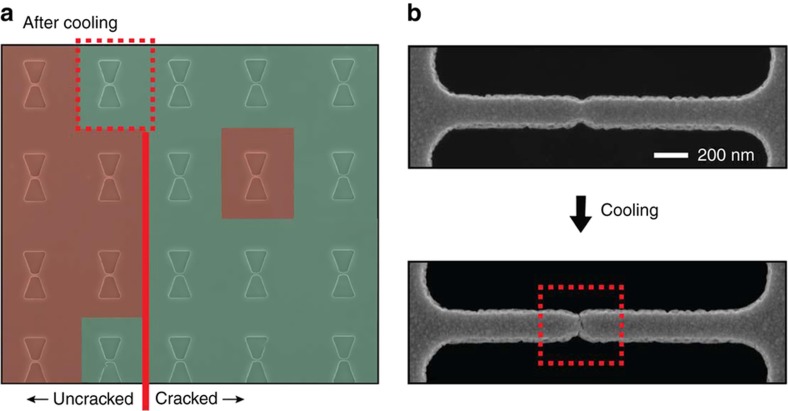
(**a**) Color-coded SEM images with top view of the same crack-junction (CJ) matrix shown in Figure 4, taken after cryogenic cooling of the chip to 77 K. A previously uncracked electrode-bridge fractured during the cooling procedure. (**b**) SEM images with top views of a representative CJ that was formed by cryogenic cooling of the substrate.

We have carried out cooling experiments using the chip containing the CJ matrix shown in Figure 4 used to explore the impact of the notch design on the formation of the crack in the electrode-bridges. We observed that, after cooling the chip to 77 K using liquid nitrogen as cooling agent, it was possible to crack one of the electrode-bridges that was uncracked prior to cooling, as shown in Figure 9a. This two-step fabrication scheme for forming cracked electrode-bridges allows the final electrode formation step to be executed inside an inert atmosphere or a vacuum, instead of forming the electrodes in the etching solution used to release etch the electrode-bridges. This way, nanogap electrodes featuring perfectly clean and pristine electrode surfaces can be realized.

## CONCLUSIONS

In this work, we have investigated and discussed the influence of critical design parameters in the formation of CJs and verify the design criteria for accurate prediction of crack formation in electrode-bridges featuring a beam width of 280 nm and beam lengths ranging from 1 to 1.8 μm. Our key findings are that to realize crack formation with a very high yield, the process to define the electrode-bridge dimensions must be well controlled to obtain reproducible localized stresses at the notches. It is possible to design the electrode-bridges in a way that the localized stresses exceed the fracture strength σ*_max_ of the electrode material significantly which, in practice, involves increasing the constriction effect by designing CJs with very small notched cross-sections. Crack formation can also be promoted by using an electrode layer featuring a high internal stress, which has the additional benefit of simultaneously decreasing the overall footprint of the CJs since, for a given gap-width, the electrode-bridge can be made shorter. Our work highlights that for predicting the resulting gap-width of a CJ, the contributions to the electrode displacements caused by the anchor overhangs have to be accounted for as they can easily increase the width of the resulting nanogap by a few nanometers. We also observe that the smearing of sharper features for long electrode-bridges can affect the yield of fracture significantly. Finally, we have proposed and demonstrated that the final crack formation event can be initiated in an inert atmosphere during a separate cooling step after release etching of the electrode-bridges.
