# Supplementary material for: Design and fabrication of crack-junctions
Source: Microsyst Nanoeng. 2017 Oct 23;3:17042. doi: 10.1038/micronano.2017.42 (PMC6444981; doi:10.1038/micronano.2017.42)
Supplement: Supplementary Information [file micronano201742-s1.pdf]

Supplementary file

Design and fabrication of crack-junctions

Valentin Dubois, Frank Niklaus and Göran Stemme

Microsystems & Nanoengineering (2017) **3,** 17042; doi:10.1038/micronano.2017.42; Published online: 23 October 2017

1. DESCRIPTION OF CONSTITUTIVE MODEL IN COMSOL MULTIPHYSICS

I. Geometry

- ***LiveLink for SolidWorks***:

  List of the geometrical parameters (see Figure S1):
  - Half-beam length $L/2$
  - Undercut length $U/2$
  - Half notch opening angle $\alpha/2$
  - Notch radius r
  - Notch indent t

- ***Difference*** (to obtain a quarter of a crack-junction)

II. Materials

- ***Poisson ratio***: 0.28 (in electrode and sacrificial layers)
- ***Young's modulus***: 380 GPa (in electrode layer)
- ***Young's modulus***: 170 GPa (in sacrificial layer)

III. Solid Mechanics: (see Figure S2)

- ***Linear elastic material*** (in electrode and sacrificial layers)

  - ***Initial stress and strain***: $\sigma_{0,\ 11} = \sigma_{0,\ 22} = 1$ GPa (in electrode layer only)

- ***Prescribed displacement 1*** (PD1): $u_{0x} = 0$
- ***Prescribed displacement 2*** (PD2): $u_{0y} = 0$
- ***Symmetry 1***
- ***Symmetry 2***
- ***Fixed constraint***

IV. Mesh: (see Figure S3)

- ***Size***:

  - Maximum element size: 1.75 μm
  - Minimum element size: 7.5 nm
  - Maximum element growth rate: 1.2
  - Curvature factor: 0.11
  - Resolution of narrow regions: 0.85

- ***Distribution1***:

  - Number of elements: 50 (at notch tip)

- ***Free tetrahedral***:

  - Geometric entity level: Remaining

V. Study

- ***Stationary***

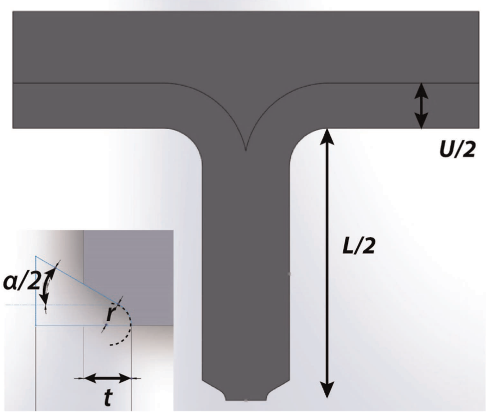

Figure S1 Bottom view of half of a crack-junction with bottom left inset of the notch as appearing in SolidWorks with parameters set in Comsol Multiphysics and synchronized with SolidWorks during simulation. The half crack-junction is split in two at its central symmetry in Comsol Multiphysics to obtain only a quarter crack-junction to simulate.

2. TITANIUM NITRIDE CRACK ROUGHNESS

In poly-crystalline titanium nitride (TiN), cracks propagate preferentially along grain boundaries[1]. Thus, the resulting cracked TiN surfaces feature jagged surface topographies as illustrated in Figure S4. Due to the non-planar geometry of the cracked surfaces, the nanogap width is not uniform over the electrode

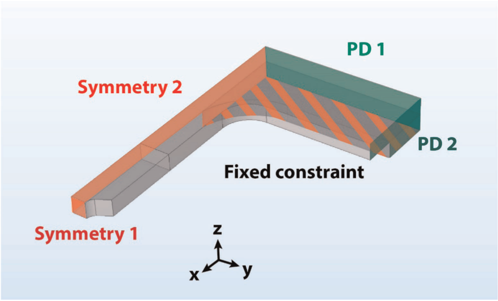

Figure S2 Depiction of boundary conditions used in Comsol Multiphysics for quarter of a crack-junction.

KTH Royal Institute of Technology, Micro and Nanosystem, Osquldas väg 10, Stockholm 100 44, Sweden
Correspondence: Valentin Dubois (valentin.dubois@ee.kth.se) or Frank Niklaus (frank.niklaus@ee.kth.se)

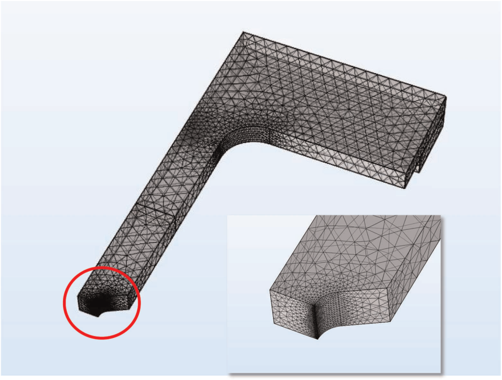

Figure S3 Complete mesh of a quarter of a crack-junction as appearing in Comsol Multiphysics, containing 41 486 domain elements, 7678 boundary elements, and 599 edge elements. A mesh refining was added at the notch, which is where the maximum stress is extracted from.

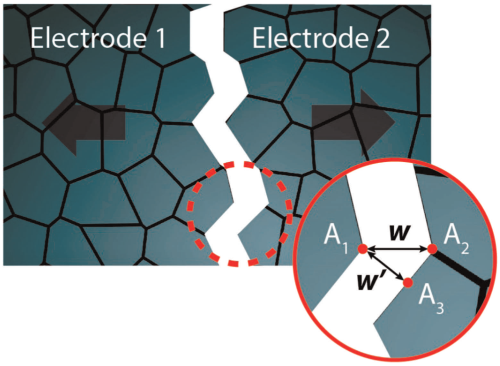

Figure S4 Impact of crack roughness on the definition of gapwidth. During crack formation, an initial point A placed precisely on the crack line is split in two corresponding points (A_1, A_2) that are displaced by w. Due to the local orientation of the crack, the shortest distance between A_1 and the left cracked surface is w', with its corresponding point A_3.

area. A point A that is placed exactly at the crack-line is split in two corresponding points A1 and A2 placed at the two electrode surfaces. Due to the contraction of the electrodes, the two corresponding points are then displaced from each other by the distance *w* in the direction parallel to the contracting direction of the electrodes, as illustrated in Figure S4 where the two black arrows indicate the direction of retraction of the cracked surfaces after crack formation. Depending on the local orientation of the crack with respect to the direction of contraction of the electrode, the resulting distance between the two electrode surfaces is smaller than *w* (e.g. A1-A3 in Figure S4). This effect can be particularly significant when the grain size is on the same order as *w* and cause significant device-to-device variability among tunneling junctions for which the electrical current depends exponentially on the inter-electrode separation[2]. However, a consequence of the perfectly matching electrode surfaces is a significantly reduced risk for unwanted electrical contact between the electrodes after crack-formation, even at very small gapwidths *w*. This is because a protrusion in one electrode surface is necessarily mirrored by a recess in the opposite electrode surface.

Regardless, crack roughness can be minimized by using electrode materials that exhibit lower (ideally amorphous) or higher (ideally single crystalline) crystallinity. Adjusting the cooling speed directly after deposition of the electrode material or adding an annealing step may be used to affect the resulting crystallinity of the electrode material and could potentially be exploited to minimize crack roughness. Using a thin film electrode material that is single crystalline would potentially produce crystallographically oriented channeling cracks and atomically flat cracked surfaces could thus be obtained. However, the use of epitaxially grown single crystalline thin films sets stringent limitations on the choice of possible electrode and sacrificial materials.

COMPETING INTERESTS

The authors declare no conflict of interest.

REFERENCES

1 Dubois V, Niklaus F, Stemme G. Crack-defined electronic nanogaps. *Advanced Materials* 2016; **28**: 2178–2182.

2 Dubois V, Niklaus F, Stemme G. Design optimization and characterization of nanogap crack-junctions. 2017 IEEE 30th International Conference on Micro Electro Mechanical Systems (MEMS); 22–26 Jan 2017; Las Vegas, NV, USA; 2017: 644-647.
